# Developmental Profile of Children Referred for Developmental Assessment Using the Developmental Assessment Scale for Indian Infants (DASII): A Retrospective Cross-Sectional Study

**DOI:** 10.7759/cureus.115827

**Published:** 2026-09-05

**Authors:** Anjali Bangalore, Madhurasree Nelanuthala, Sangita M More, Mohammad Haseeb, Balam Rishita, Mohd S Siddiqui, Anju M Varghese, Abinash Mohapatra

**Affiliations:** 1 Pediatrics and Child Health, ICON Centre for Assisted Learning, Chhatrapati Sambhajinagar, IND; 2 Pediatrics, Mahatma Gandhi Mission (MGM) Medical College and Hospital, MGM Institute of Health Sciences, Chhatrapati Sambhajinagar, IND; 3 Pediatrics, Spandana Hospital, Dharmavaram, IND

**Keywords:** developmental assessment scale for indian infants, developmental delay, developmental quotient, india, nicu, prematurity

## Abstract

Introduction: Developmental delay is a major pediatric concern, and children born preterm or requiring neonatal intensive care are at particularly increased risk. The Developmental Assessment Scale for Indian Infants (DASII) is a validated tool for evaluating motor and mental development in children up to 30 months of age, but detailed profiles of children referred for suspected developmental delay in Indian clinical settings remain sparse. This study aimed primarily to describe the developmental profile of children referred for developmental assessment using the DASII. As a secondary exploratory analysis, developmental measures were compared between term and preterm children, with recognition of the unstratified gestational grouping and potential differences in referral pathways and clinical characteristics.

Methods: This retrospective cross-sectional study analyzed medical records of 202 children aged 3-30 months who underwent DASII assessment between January 2023 and December 2025 at a developmental assessment center in Chhatrapati Sambhajinagar, Maharashtra, India. Motor and mental developmental quotients (Developmental Motor Quotient (DMoQ) and Developmental Mental Quotient (DMeQ)) and developmental ages were recorded. Outcomes were compared between term and preterm children using Student's t-test/Mann-Whitney U test and chi-square/Fisher's exact test, as appropriate.

Results: Among 202 children assessed using DASII, motor developmental delay was present in 90.6% and mental developmental delay in 92.1%, with combined motor and mental delay being the predominant pattern (86.1%). In the exploratory term-preterm analysis, selected developmental scores and developmental ages differed between groups; however, DMoQ and DMeQ did not differ significantly after adjustment for multiple comparisons. A higher proportion of preterm children had no categorical mental developmental delay than term children; however, this finding should be interpreted cautiously given the referral-based design, unstratified gestational grouping, and small preterm subgroup.

Conclusion: Children referred for developmental assessment demonstrated a high burden of developmental impairment, with combined motor and mental delay being the predominant pattern. Differences between term and preterm children varied depending on the developmental measure used and should therefore be interpreted with caution. Prospective studies with larger sample sizes and comprehensive domain-specific assessments are warranted.

## Introduction

Developmental delay is a major public health concern and a leading cause of childhood disability worldwide [1]. The burden is particularly high in low- and middle-income countries, where biological, perinatal, nutritional, and environmental risk factors adversely affect early childhood development [1]. Accurate developmental assessment is fundamental for the early detection and monitoring of developmental disorders. In India, the Developmental Assessment Scale for Indian Infants (DASII) is one of the most widely used standardized tools for assessing neurodevelopment in children up to 30 months of age [2]. Developed as an Indian adaptation of the Bayley Scales of Infant Development [3], DASII provides quantitative assessment of motor and mental development through the Developmental Motor Quotient (DMoQ) and Developmental Mental Quotient (DMeQ), and can additionally evaluate performance across specific developmental domains when such data are available [4]. In clinical practice, a developmental delay is typically defined as a developmental quotient (DQ) of less than 70, representing scores two standard deviations (SDs) below the mean [2]. DASII has been extensively utilized for developmental surveillance of high-risk infants, including preterm neonates, neonatal intensive care unit (NICU) graduates, and children with various perinatal risk factors [5-8].

Although several Indian studies have evaluated developmental outcomes among high-risk infants, very few have described the developmental profile of children referred specifically for developmental evaluation in routine clinical practice using DASII. The primary objective of this study was to describe the developmental profile of children referred for developmental assessment using the DASII, including the frequency and patterns of motor and mental developmental delays. As a secondary exploratory objective, developmental measures were described according to term and preterm status.

## Materials and methods

Study design and setting

This retrospective cross-sectional observational study was conducted at the ICON Learning Center, a developmental assessment center located in Chhatrapati Sambhajinagar (Aurangabad), Maharashtra, India. Medical records of children who underwent developmental assessment between January 2023 and December 2025 were reviewed.

Study population

The study included records of 202 children aged 3-30 months who were referred for developmental evaluation during the study period. Children were referred to the developmental assessment center through clinical referrals for suspected developmental concerns. The retrospective records did not consistently document whether a referral was initiated due to overt developmental concerns, parental concerns, or proactive high-risk neonatal surveillance. Because referral indications and pathways were not systematically coded in the retrospective database, differences in referral ascertainment between term and preterm children could not be quantified. A formal sample size calculation was not performed; 202 was a convenience sample of available records from the study window. Children with complete developmental assessment data available in the medical records were included in the analysis. Of the 215 records screened, 13 were excluded due to incomplete documentation/missing developmental assessment data, leaving 202 records for analysis.

Developmental assessment

Development was assessed using the DASII, a standardized developmental assessment tool developed by Phatak et al. for Indian children aged 0-30 months [2,4]. All developmental assessments were performed by a certified developmental pediatrician trained in administering the DASII. Delay was defined as a development quotient (DQ) score of less than 70 (which is two SDs below the mean) on either the mental or motor scale [2]. Gestational status was categorized as term (≥37 completed weeks) or preterm (<37 completed weeks). Corrected age was used for developmental assessment in preterm children; corrected age was calculated by subtracting the number of weeks born before 40 weeks gestation from chronological age, while chronological age was recorded for all participants. Because detailed gestational-age strata and neonatal injury phenotypes were not consistently available in the retrospective records, the term-preterm comparison was considered a secondary exploratory analysis rather than a primary clinical hypothesis.

Data collection

Demographic and clinical information was extracted from medical records, including age at assessment, sex, gestational status (term or preterm), mode of delivery, NICU admission, indication for NICU admission, and developmental assessment findings. Both chronological age and corrected age (for preterm infants) were documented.

Statistical analysis

Data were entered into a structured database, and statistical analyses were performed using IBM Statistical Package for the Social Sciences Statistics for Windows, version 24.0 (IBM Corp., Armonk, NY). Continuous variables were summarized as mean ± SD or median with interquartile range, depending on data distribution. Categorical variables were expressed as frequencies and percentages.

Motor and mental developmental outcomes were described using DMoQ, DMeQ, and developmental age scores. Comparisons between term and preterm children were performed using Student's t-test or Mann-Whitney U test for continuous variables and chi-square test or Fisher's exact test for categorical variables, as appropriate. A p value of less than 0.05 was considered statistically significant. For the exploratory comparisons of multiple developmental outcomes between term and preterm children, p values were adjusted using the Holm method to control the family-wise error rate. Both raw and Holm-adjusted p values are reported.

Ethical considerations

The study was conducted using anonymized retrospective medical record data. Approval from the Institutional Ethics Committee was obtained (Approval No. MGM- ECRHS/2026/100).

## Results

A total of 202 children aged 3-30 months were included in the study. The mean chronological and corrected ages were 14.6 ± 7.2 and 14.2 ± 7.3 months, respectively, and 135 (66.8%) were male children. Most children were born at term (154, 76.2%), while 48 (23.8%) were preterm. A history of NICU admission was present in 111 (55%) children, with prematurity being the most common indication (Table 1).

**Table 1:** Sociodemographic characteristics Percentages are calculated among NICU-admitted children (n = 111) ^*^The primary reason for NICU admission SD: standard deviation; NICU: neonatal intensive care unit

Characteristic	Value
Chronological age (mean ± SD	14.6 ± 7.2
Corrected age (months), (mean ± SD)	14.2 ± 7.3
Sex, n (%)	
Male	135 (66.8)
Female	67 (33.2)
Gestational status, n (%)	
Term	154 (76.2)
Preterm	48 (23.8)
Mode of delivery, n (%)	
Normal vaginal delivery	113 (55.9)
Cesarean section	89 (44.1)
NICU admission, n (%)	111 (55)
No NICU admission, n (%)	91 (45)
Major indication for NICU admission^*^, n (%)	
Prematurity	47 (42.3)
Birth asphyxia	26 (23.4)
Neonatal seizures	19 (17.1)
Neonatal sepsis	8 (7.2)
Neonatal hyperbilirubinemia	6 (5.4)
Meconium aspiration syndrome	4 (3.6)
Intrauterine growth restriction	1 (0.9)

Table 2 presents the developmental assessment scores of the study participants. The median motor score was 34.5 (Q1-Q3: 26.0-43.0), with a median DMoQ of 55.5 (38.0-70.75). The median motor developmental age at the 50th percentile was 5.7 months (4.6-8.7). Regarding mental development, the median mental score was 79.0 (59.0-100.75), the median DMeQ was 56.0 (40.25-68.0), and the median mental developmental age at the 50th percentile was 6.25 months (4.4-10.7). Motor developmental delay was observed in 183 (90.6%) participants, whereas only 19 (9.4%) participants did not exhibit motor delay. Similarly, mental developmental delay was present in 186 (92.1%) participants, while 16 (7.9%) participants had no evidence of mental delay.

**Table 2:** Developmental assessment scores of study participants Continuous variables are presented as median (Q1-Q3) because of nonnormal distribution. Categorical variables are presented as frequency (percentage). Developmental delay was defined according to DASII criteria DASII: Developmental Assessment Scale for Indian Infants

Variable	Median (Q1-Q3)
Motor score	34.5 (26.0-43.0)
Developmental Motor Quotient	55.5 (38.0-70.75)
Motor developmental age (50th percentile), months	5.7 (4.6-8.7)
Motor developmental delay, n (%)	183 (90.6%)
No motor developmental delay, n (%)	19 (9.4%)
Mental score	79.0 (59.0-100.75)
Developmental Mental Quotient	56.0 (40.25-68.0)
Mental developmental age (50th percentile), months	6.25 (4.4-10.7)
Mental developmental delay, n (%)	186 (92.1%)
No mental developmental delay, n (%)	16 (7.9%)

Among the 202 children assessed using DASII, combined motor and mental developmental delay (global developmental delay) was the most common pattern, observed in 174 (86.1%) children. Isolated motor delay was identified in nine children (4.5%), while isolated mental delay was present in 12 children (5.9%). Only seven children (3.5%) demonstrated normal motor and mental developmental outcomes (Table 3). Among the 183 children with motor delay, 174 (95.1%) also had mental delay. Similarly, among the 186 children with mental delay, 174 (93.5%) had concomitant motor delay. Fisher's exact test demonstrated a significant association between motor and mental developmental delay (Fisher's exact test, p < 0.001) (Table 4).

**Table 3:** Association statistics for motor and mental developmental delay n/N represents the number with outcome/subgroup denominator (183 children with motor delay; 186 children with mental delay). Fisher's exact test was used to assess the association between motor and mental developmental delay

Measure	Value
Motor delay with concomitant mental delay, n/N (%)	174/183 (95.1)
Mental delay with concomitant motor delay, n/N (%)	174/186 (93.5)
Fisher's exact test, p value	<0.001

**Table 4:** Association between motor and mental developmental status among children assessed using DASII (N = 202) Percentages are expressed as a proportion of the total study population (N = 202) DASII: Developmental Assessment Scale for Indian Infants

Motor developmental status	Mental delay, n (%)	No mental delay, n (%)	Total, n (%)
Motor delay	174 (86.1)	9 (4.5)	183 (90.6)
No motor delay	12 (5.9)	7 (3.5)	19 (9.4)
Total	186 (92.1)	16 (7.9)	202 (100)

Tables 5, 6 show developmental outcomes between term and preterm children. Preterm children had lower median raw motor and mental scores and lower motor and mental developmental ages than term children. However, DMoQ did not differ significantly between term and preterm children (median 53.0 vs. 65.5, p = 0.188), and DMeQ also did not differ significantly (median 55.0 vs. 65.0, p = 0.062). Regarding categorical developmental status, the proportion of children without mental developmental delay was significantly higher among preterm children than term children (10/48 (20.8%) vs. 6/154 (3.9%); Fisher's exact p < 0.001).

**Table 5:** Exploratory comparison of motor and mental developmental delay between term and preterm children Values are presented as frequencies (percentages). Percentages are calculated within each gestational group (term or preterm). Comparisons between groups were performed using Pearson's chi-square test or Fisher's exact test, as appropriate

Variable	Term (n = 154)	Preterm (n = 48)	Statistical test	p value
Motor developmental delay, n (%)				
Delay	143 (92.9)	40 (83.3)	Fisher's exact test	0.069
No delay	11 (7.1)	8 (16.7)	-	-
Mental developmental delay, n (%)				
Delay	148 (96.1)	38 (79.2)	Fisher's exact test	<0.001
No delay	6 (3.9)	10 (20.8)	-	-

**Table 6:** Exploratory comparison of developmental assessment scores between term and preterm children Continuous variables are presented as the first quartile (Q1), median, and third quartile (Q3). Comparisons between term and preterm children were performed using the Mann-Whitney U test because the data were not normally distributed. Because multiple developmental outcomes were compared, p values were adjusted using the Holm method. A two-sided adjusted p value <0.05 was considered statistically significant

Variable	n	Term (Q1, median, Q3)	Preterm (Q1, median, Q3)	Statistical test	p value	Holm-adjusted p value
Motor score	202	28.0, 36.0, 43.0	22.0, 28.0, 43.0	Mann-Whitney U	0.012	0.042
Developmental Motor Quotient	202	38.0, 53.0, 69.0	34.8, 65.5, 77.0	Mann-Whitney U	0.188	0.188
Motor developmental age (50th percentile)	199	4.9, 6.6, 8.7	3.5, 4.8, 8.7	Mann-Whitney U	0.005	0.031
Mental score	202	62.0, 81.0, 104.8	48.8, 67.5, 92.5	Mann-Whitney U	0.008	0.042
Developmental Mental Quotient	202	41.3, 55.0, 65.0	35.3, 65.0, 85.5	Mann-Whitney U	0.062	0.124
Mental developmental age (50th percentile)	198	4.7, 6.4, 11.4	3.4, 5.2, 9.5	Mann-Whitney U	0.010	0.042

## Discussion

In this retrospective study of 202 children referred for developmental assessment, a high proportion demonstrated developmental delay on DASII evaluation, with combined motor and mental delay constituting the predominant pattern of impairment. These findings highlight the substantial burden of developmental impairment among children referred for specialized developmental evaluation in this clinical setting. However, because the cohort was referral-based, these findings should not be interpreted as estimates of developmental delay in the general pediatric population.

The mean corrected age at assessment was 14.2 ± 7.3 months. This age corresponds to a period when gross motor milestones, including independent standing and initiation of walking, become increasingly observable to caregivers and clinicians and may contribute to referral for developmental assessment. Although assessment during this period permits early identification and intervention, delayed referral may reflect limited parental awareness, delayed recognition of subtler developmental concerns, or restricted access to specialized developmental assessment services. The cohort also demonstrated a male predominance (66.8%), consistent with previous Indian clinic-based cohorts evaluated for developmental and neurodevelopmental disorders [9,10]. Given the referral-based nature of the cohort, this pattern may reflect differences in healthcare-seeking or referral behavior rather than a true difference in the underlying prevalence of developmental delay.

More than half of the children had a history of NICU admission, with prematurity being the most frequent indication, followed by birth asphyxia, neonatal seizures, neonatal sepsis, neonatal jaundice, meconium aspiration syndrome, and intrauterine growth restriction. These conditions have been associated with adverse neurodevelopmental outcomes in previous studies [11-15]. However, their individual associations with developmental outcomes could not be examined in the present retrospective dataset because the study was primarily designed to describe the developmental profile of the referred cohort.

A high proportion of children exhibited both motor and mental developmental delay, which is expected in a referral-based cohort enriched for children with suspected developmental concerns. The observed prevalence of developmental delay was therefore substantially higher than that reported in community-based studies of unselected Indian children, where estimates are generally lower [15]. These findings should be interpreted as describing the developmental profile of a clinically referred population rather than as population-level estimates. Combined motor and mental developmental delay was markedly more common than isolated motor or isolated mental delay, suggesting that developmental difficulties in this referred population were frequently multidomain rather than restricted to a single developmental domain.

Motor and mental developmental delay were strongly associated (Fisher's exact test, p < 0.001). This association may reflect overlapping effects of perinatal insults, shared developmental processes assessed by the two DASII domains, and referral of children with more global developmental concerns. Isolated or milder developmental difficulties may also be less likely to result in referral for comprehensive developmental assessment. Regardless of the underlying mechanism, these findings highlight the potential limitation of relying exclusively on isolated developmental milestones or single-domain screening. Comprehensive developmental assessment may help identify children with broader developmental difficulties who require multidisciplinary evaluation and intervention.

The comparison between term and preterm children was performed as a secondary exploratory analysis. Selected raw developmental scores and developmental ages differed between the groups; however, DMoQ and DMeQ did not differ significantly after adjustment for multiple comparisons. Conversely, a higher proportion of preterm children were classified as having no categorical mental developmental delay compared with term children (20.8% vs. 3.9%; Fisher's exact p < 0.001). This finding should not be interpreted as evidence of better mental development among preterm children. Differences in referral and ascertainment pathways may have influenced the observed comparison; for example, preterm children may have undergone developmental assessment through high-risk surveillance, whereas term children may have been referred predominantly because of recognized developmental concerns. However, referral indications were not systematically coded in the retrospective database, and this possibility could not be directly evaluated.

Furthermore, all children born before 37 weeks were analyzed as a single preterm group. The preterm subgroup therefore included children with potentially different degrees of prematurity and different neonatal clinical courses. Gestational-age stratification and characterization of neonatal neurological injury were not possible because these data were not consistently available. In addition, developmental assessment in preterm children was based on corrected age, and differences in age correction and developmental timing should be considered when interpreting direct comparisons of DQs between term and preterm children. The relatively small preterm subgroup (n = 48) further limits the precision of these exploratory comparisons. Accordingly, the term-preterm findings should be regarded as descriptive observations rather than evidence supporting a specific biological hypothesis or causal effect of prematurity on developmental outcomes.

The findings support the importance of structured developmental surveillance and timely comprehensive assessment for children with recognized biological or clinical risk factors, including preterm birth or a history of NICU admission. Standardized instruments such as DASII can facilitate systematic identification of developmental difficulties and support timely referral to early intervention services. However, the present study was not designed to evaluate the effectiveness of specific interventions or developmental outcomes following intervention.

The strengths of this study include its relatively large sample size for a single-center developmental assessment cohort, the use of a standardized developmental assessment instrument, and the uniform administration of the DASII by a single trained developmental pediatrician. These features provided consistency in the assessment process. The study also provides clinical data from a referral-based developmental assessment setting, which may be useful for understanding the pattern of developmental difficulties encountered in routine clinical practice.

This study has several limitations. Its retrospective, single-center, referral-based design limits generalizability and precluded assessment of longitudinal developmental trajectories or intervention outcomes. The absence of a control group and lack of socioeconomic, maternal, and environmental variables prevented normative or adjusted comparisons. The preterm subgroup was relatively small (n = 48) and was analyzed as a single group without reliable gestational-age stratification or systematic information on neonatal neurological and illness-severity markers, including neuroimaging findings, duration of invasive ventilation, necrotizing enterocolitis, and neonatal sepsis. Differences in referral and ascertainment pathways between term and preterm children may also have influenced the observed group differences. Corrected age was used for preterm children, which should be considered when interpreting direct comparisons of DQs. Finally, although all assessments were performed by a single trained developmental pediatrician, providing consistency in administration, interrater reliability could not be assessed, and observer or expectancy bias cannot be excluded. Detailed DASII subdomain data were also not consistently available in the retrospective records. The term-preterm comparisons were exploratory and were interpreted with adjustment for multiple testing.

Future prospective, multicenter studies with adequate sample sizes, gestational-age stratification, comprehensive neonatal and sociodemographic data, complete DASII domain-level information, and longitudinal follow-up are warranted to better characterize developmental trajectories and clarify developmental outcomes across different preterm subgroups.

## Conclusions

Children referred for developmental assessment demonstrated a high burden of developmental impairment, with combined motor and mental delay being the predominant pattern. The term-preterm comparisons were exploratory and should not be interpreted as evidence of a causal effect of prematurity because the preterm group was not stratified by gestational age or neonatal clinical phenotype, and referral pathways may have differed between the groups. Prospective studies with larger, gestational-age-stratified cohorts and comprehensive neonatal and socioeconomic data are needed to clarify developmental outcomes across preterm subgroups.
